# Dietary Chitosan Oligosaccharide Supplementation Improves Meat Quality by Improving Antioxidant Capacity and Fiber Characteristics in the Thigh Muscle of Broilers

**DOI:** 10.3390/antiox13030366

**Published:** 2024-03-18

**Authors:** Ruixia Lan, Yuchen Wang, Haoxuan Wang, Jia Zhang

**Affiliations:** Department of Animal Science and Technology, College of Coastal Agriculture Sciences, Guangdong Ocean University, Zhanjiang 524088, China; wangyuchen@stu.gdou.edu.cn (Y.W.); 2112204086@stu.gdou.edu.cn (H.W.); 2112204108@stu.gdou.edu.cn (J.Z.)

**Keywords:** meat quality, fatty acids, antioxidant capacity, myofiber characteristics, chitosan oligosaccharides, broilers

## Abstract

This study investigated the effects of dietary chitosan oligosaccharide (COS) supplementation on meat quality, antioxidant capacity, and muscle fiber characteristics in the thigh muscle of broilers. The results showed that dietary COS supplementation decreased shear force and increased crude protein content and nutritional value in the thigh muscle, while decreasing the content of C16:0, C18:0, and total saturated fatty acids. Dietary COS supplementation increased free radical scavenging activity, antioxidant enzyme activity, and antioxidant enzyme-related gene expression. Additionally, COS promoted *MyHCI* while decreasing *MyHCIIb* mRNA expression levels. The myofiber transformation was associated with upregulated gene expression of *CaN*, *NFATc1*, *MyoD*, and *SIRT1.* Together, the results of this study demonstrate that dietary COS supplementation improves meat quality, nutritional value, antioxidant capacity, and myofiber transformation to more oxidative muscle fibers in the thigh muscle of broilers when its supplemental level is 400 mg/kg.

## 1. Introduction

Chicken meat is considered a high-quality meat due to its favorable nutritional composition, which includes high protein and unsaturated fatty acids, as well as low saturated fatty acids. In 2020, of all the world’s meat types, chicken meat production reached the highest proportion (35.44%) due to its high nutritional content and affordable price [1]. With improving living standards, consumers demand higher quality chicken meat products. Currently, ensuring meat quality is the most important challenge faced in the poultry industry, as poor meat quality decreases consumers’ acceptance and financial profitability [2,3]. Chicken meat quality is affected by many factors, including the chicken breed, management, rearing conditions, feed composition, and nutrition. In terms of nutritional manipulation, certain dietary feed additives can directly influence meat quality and antioxidant capacity in the breast and thigh muscle of broilers [4]. These findings suggest that nutritional manipulation is an effective strategy to enhance meat quality in broilers. Along these lines, the use of natural antioxidants is popular in industry and among consumers.

Chitosan oligosaccharides (COS) have been reported to exhibit various biological properties, including free radical scavenging capacity and antioxidant, anti-inflammation, immune modulation, and anti-bacterial effects, and they are widely used in animal production and health care [5,6,7]. Hence, COS are considered a potential feed additive to enhance meat quality in broilers [8].

Chicken meat quality is directly associated with antioxidant capacity, myofiber composition, and the proportion of myofiber types [9,10]. Oxidative stress in muscle leads to damage to both meat quality and antioxidant systems, which is highly associated with the overproduction of reactive oxygen species (ROS), lipid oxidation, decreased antioxidant enzyme activity, and antioxidant enzyme-related gene expression [8,11]. Numerous studies have reported that COS had beneficial effects on meat quality and muscle antioxidant capacity in broilers, and the better meat quality was associated with better antioxidant status in muscle [8,12,13,14,15]. Poultry muscle is highly sensitive to oxidative damage owing to its high content of unsaturated fatty acids [16]. In addition, the composition and proportion of myofiber types are directly associated with meat quality, including pH, meat color, water holding capacity, and flavor [9,17,18]. In poultry, according to the myosin heavy chain (*MyHC*) polymorphism, myofibers are divided into three types: *MyHCI* (slow-red-twitch fiber, slow oxidative), *MyHCIIa* (fast-red-twitch fiber, fast oxidative), and *MyHCIIb* (fast-white-twitch fiber, fast glycolytic) [19]. Studies in pigs have demonstrated that feed additive supplementation could improve pork quality by inducing myofiber type conversion and altering myofiber type compositions and proportions [17,18]. Dietary carnosine supplementation improved the thigh muscle meat quality in broilers, which was associated with the myofiber type conversion and composition, as well as better antioxidant capacity [9]. Overall, meat quality is connected with antioxidant capacity and myofiber characteristics [18,20]. Although our former study indicated that COS supplementation could enhance meat quality in broilers, there is little information available concerning the effects of COS supplementation on myofiber type conversion. Relatively few studies have been conducted to evaluate the effects of COS supplementation on meat quality in systematic way, let alone using a broiler model. Therefore, we hypothesize that COS supplementation improves meat quality by enhancing antioxidant capacity and regulating myofiber type conversion. To verify this hypothesis, this study was firstly conducted to investigate the effects of different levels of COS supplementation on meat quality, antioxidant status, and myofiber characteristics in the thigh muscle of broilers.

## 2. Materials and Methods

### 2.1. Experimental Design, Housing, and Diets

The Animal Care and Use Committee of Guangdong Ocean University, China (SYXK-2018-0147) approved the use of broilers and experimental protocols. In total, 240 one day-old male Arbor Acres broilers with an initial body weight of 40.47 ± 0.30 g were randomly divided into four groups with six replicate cages, and each replicate cage had ten broilers. The broilers were fed a corn–soybean meal basal diet supplemented with 0, 200, 400, and 800 mg/kg of COS (CON group, COS_200_ group, COS_400_ group, and COS_800_ group). The basal diet was formulated to meet or exceed the National Research Council (NRC, 2012) nutrient requirements, and is listed in Supplemental Table S1. The COS used in this study were provided by Qindao Songtian Biotechnology Co., Ltd. (Qindao, China), and the purity of the COS was over 90%, with an average molecular weight less than 3000 Da and a deacetylation degree greater than 90.00%. The basal diet was first prepared, then divided into five equal portions to facilitate the COS supplementation.

### 2.2. Sample Collections

On day 42, after overnight fasting, six broilers from each treatment (one broiler per cage) were selected, weighed individually, and sacrificed. The left thigh muscle samples (M. iliotibialis cranialis) were collected into RNase free tubes, immediately frozen in liquid nitrogen, and stored at −80 °C to determine fatty acid compositions, antioxidant status, and related gene expression. About 1 cm^3^ thigh muscle samples (M. iliotibialis cranialis) were collected and fixed in 4% paraformaldehyde for hematoxylin and eosin (H.E) staining. Meanwhile, the right thigh muscle samples (M. iliotibialis cranialis) were collected and stored at 4 °C for 24 h to evaluate the meat quality and nutrient composition.

### 2.3. Meat Quality and Nutrient Composition

Meat quality and nutrient composition measurement following the methods described in our former study [12]. Briefly, triplicate pH was collected 45 min (pH_45min_) and 24 h (pH_24h_) post-mortem using a pH meter (AZ8694, Taiwan Hengxin Co., Ltd., Shenzhen, China). Meat color was measured at 24 h post-mortem with a CR-10 Plus Chroma meter (Konica Minolta Sensing Inc., Osaka, Japan). The lightness (L*), redness (a*), and yellowness (b*) were measured from three different locations. About 4 g samples were packaged in plastic bags and stored at 4 °C for 24 h, then the samples were reweighed to calculate the drip loss. For cooking loss determination, about 4 g samples were weighed, placed in a plastic bag, and cooked in a water bath at 75 °C until the internal temperature reached 72 °C; then, they were cooled at room temperature for 30 min, blotted dry, and weighed to calculate cooking loss. Shear force was measured after cooking loss, and the cooked samples were cut perpendicular to the fiber orientation using a texture analyzer (Bulader-TS100, Beijing Bulader Co., Ltd., Beijing, China).

For muscle nutrient composition analysis, about 40–50 g fresh thigh muscle samples were weighed, sliced up, placed in weighting bottles, and dried at 65 °C for 72 h in a dryer and reweighed. The dry matter was calculated using the difference between the initial and the dried sample weights. Then, the dried muscle samples were ground into powder to analyze the crude protein and crude fat contents. The crude protein (CP, 991.36) and crude fat (EE, 991.36) content analysis following the methods described by the AOAC [21].

### 2.4. Fatty Acid Composition and Nutritional Value

Fatty acid composition determination followed the methods described by Wassie et al. [22]. Briefly, the one-step method for methyl ester was used to extract lipids from freeze-dried thigh muscle, then the supernatant was filtered and analyzed using a gas chromatograph (Agilent-8890, Agilent Technologies Inc., Santa Clara, CA, USA). The same determination was made with the standard sample. Each fatty acid content was obtained using a standard curve, and its composition was measured using the area normalization method and expressed as ug/g.

The fatty acids were divided into saturated fatty acids (SFAs, including C6:0, C8:0, C10:0, C12:0, C13:0, C14:0, C15:0, C16:0, C17:0, C18:0, C19:0, C20:0, C21:0, and C22:0), monounsaturated fatty acids (MUFAs, include C14:1, C16:1, C17:1, C18:1n9c, C20:1n9, and C24:1n9), polyunsaturated fatty acids (PUFAs, include C18:2n6, C18:3n6, C18:3n3, C20:2, C20:3n6, C20:3n3, C20:4n6, C22:5n3, and C22:6n3), total n-6 PUFAs (including C18:2n6, C18:3n6, C20:3n6, and C20:4n6), and total n-3 PUFAs (including C18:3n3, C20:3n3, C22:5n3, and C22:6n3). To evaluate the nutrition value of thigh muscle, the nutritional value indexes were calculated using the following formulas [23]: DHA (docosahexaenoic acid) + EPA (eicosatetraenoic acid) = C22:6n3 + C20:5n3; fatty acids unsaturation index (UI) = percentage of monoenoic acid × 1 + (percentage of dienoic × 2) + (percentage of trienoic × 3) + (percentage of tetraenoic × 4) + (percentage of pentaenoic × 5) + (percentage of hexaenoic × 6); peroxidation trend index (PI) = (0.025 × percentage of monoenoic acid) + (1 × percentage of dienoyl acid) + (2 × percentage of trienoic acid) + (4 × percentage of tetraenoic acid) + (6 × percentage of pentaenoic acid) + (8 × percentage of hexanoic acid); nutrition value index (NVI) = (C18:0 + C18:1n9)/(C16:0); health-promoting index (HPI) = (ΣUFA)/(4 × C14:0 + C16:0); index of atherogenicity (IA) = (4 × C14:0 + C16:0)/(ΣMUFA + ΣPUFA); index of thrombogenicity (IT) = (C14:0 + C16:0 + C18:0)/[(0.5 × MUFA) + (0.5 × Σn-6PUFA) + (3 × Σn-3PUFA) + (Σn-3PUFA/Σn-6PUFA)]; hypocholesterolemic to hypercholesterolemic ratio (HH ratio) = (C18:1 + ΣPUFA)/(C14:0 + C16:0); and health-promoting index, (HPI) = (ΣUFA)/(4 × C14:0 + C16:0).

### 2.5. Antioxidant Status

The muscle samples were ground with liquid nitrogen, then diluted with an ice-cold physiological saline solution at a weight to volume ratio of 1:9 and centrifuged at 4200× *g* for 10 min at 4 °C to collect the supernatant. Nanjing Jiancheng commercial assay kits (Nanjing, China) were used to evaluate the antioxidant-related parameters, including oxygen radicals (O_2_·^−^), hydroxyl radicals (OH·^−^), 2, 2′-azino-bis (3-ethylbenzothiazoline-6-sulfonate) (ABTS·), malondialdehyde (MDA), glutathione peroxidase (GSH-Px), total superoxide dismutase (T-SOD), and catalase (CAT), as well as the protein level, according to the manufacturer’s instructions. The kit information is listed in Supplemental Table S2.

### 2.6. Myofiber Morphological Characteristics

Myofiber morphological characteristics were determined using H.E staining. After fixing samples in 4% paraformaldehyde for 24 h and embedding them in paraffin, a microtome was used to prepare 4 μm serial tissue sections, which were mounted on slides. Then, the samples were deparaffinized, rehydrated, rinsed in distilled water, stained with H.E, dehydrated, and mounted. The myofiber cross-sectional area (CSA) was measured using Image-Pro Plus software (Version 6.0), and the fiber diameter was calculated following the following formula: fiber diameter = 2 × (fiber cross-sectional area/π)^0.5^. For each sample, six images containing approximately 200 myofibers were measured.

### 2.7. Real-Time Quantitative PCR

Total RNA extraction followed the Trizol reagent kit’s instruction. RNA was then quantified using a UV spectrophotometer at 260 and 280 nm absorbance ratios, and the RNA integrity was verified by 1.5% agarose gel electrophoresis. After that, a one-step gDNA removal kit was used to synthesize cDNA, and a real-time quantitative polymerase chain reaction (RT-PCR) was performed as in our previously described method [24]. The housekeeping gene β-actin used as an internal reference gene, and the 2^−ΔΔCt^ method was used to calculate the relative target gene expression [25]. The primer sequences are listed in Supplemental Table S3, respectively.

### 2.8. Statistical Analysis

The pens used as the experiment units and all data were analyzed with SAS 2003 (v. 9.1, SAS Institute Inc., Cary, NC, USA) using the mixed procedure. One-way analysis of variance followed by Duncan’s multiple range test was used to evaluate statistical differences among the treatments. Unpaired Student’s *t*-tests were used to evaluate statistical differences in fatty acid compositions between the CON and COS_400_ groups. *p* < 0.05 was considered to indicate significant differences.

## 3. Results

### 3.1. Meat Quality and Nutrient Composition

In the thigh muscle (Table 1), there were no significant differences in pH_45min_, pH_24h_, L*, a*, b*, cooking loss, or crude lipids among the treatments. Compared to the CON group, dietary 200 and 400 mg/kg COS decreased (*p* < 0.05) shear force. Dietary 200 and 800 mg/kg COS decreased (*p* < 0.05) dry matter content when compared to the CON and COS_400_ groups. Dietary 400 mg/kg COS increased (*p* < 0.05) crude protein content when compared to the other groups. In addition, the drip loss in the COS_200_ and COS_400_ groups was lower than that in the COS_800_ group, while the shear force in the COS_400_ group was lower than that in the COS_800_ group. These results suggest that the best beneficial effects of COS on meat quality and nutrient composition appear when its supplemental level is 400 mg/kg.

### 3.2. Lipid Metabolism-Related Gene Expression

**Figure 1:**
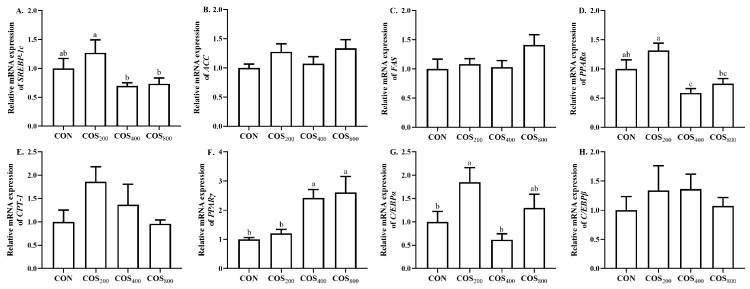
Effects of chitosan oligosaccharide supplementation on lipid metabolism-related gene expression in the thigh muscle of broilers. The relative mRNA expression of *SREBP-1c* (**A**), *ACC* (**B**), *FAS* (**C**), *PPARα* (**D**),*CPT-1* (**E**), *PPARγ* (**F**), *C/EBPα* (**G**), and *C/EBPβ* (**H**). CON, COS_200_, COS_400_, COS_800_, basal diet with chitosan oligosaccharide at 0, 200, 400, or 800 mg/kg, respectively. *SREBP-1c*, sterol regulatory element-binding protein-1c; *ACC*, acetyl-CoA carboxylase; *FAS*, fatty acid synthase; *PPAR*, peroxisome proliferator-activated receptor; *CPT-1*, carnitine palmitoyl transferase 1; *C/EBP*, CCAAT/enhancer binding protein. ^a–c^ Different superscripts indicate significant difference at *p* < 0.05.

To evaluate whether COS regulates lipid metabolism in thigh muscle, we detected the mRNA expression of lipid metabolism-related genes (Figure 1). Compared to the CON group, dietary 400 mg/kg COS downregulated (*p* < 0.05) the mRNA expression of peroxisome proliferator-activated receptor *α* (*PPARα*). Dietary 400 and 800 mg/kg COS upregulated (*p* < 0.05) the mRNA expression of *PPARγ* when compared to the CON and COS_200_ groups. Dietary 200 mg/kg COS upregulated (*p* < 0.05) the mRNA expression of CCAAT/enhancer binding protein *α* (*C/EBPα*) when compared to the CON and COS_400_ groups. In addition, the mRNA expression of sterol regulatory element-binding protein-1c (*SREBP-1c*) and *PPARα* in the COS_200_ group was higher than that in the COS_400_ and COS_800_ groups. These results suggest that the beneficial effects of COS in increasing fatty acid synthesis, decreasing fatty acid oxidation, and promoting intramuscular preadipocyte differentiation, maturation, and proliferation are maximized when its supplemental level is 400 mg/kg.

### 3.3. Fatty Acid Composition and Nutritional Indicators

**Figure 2:**
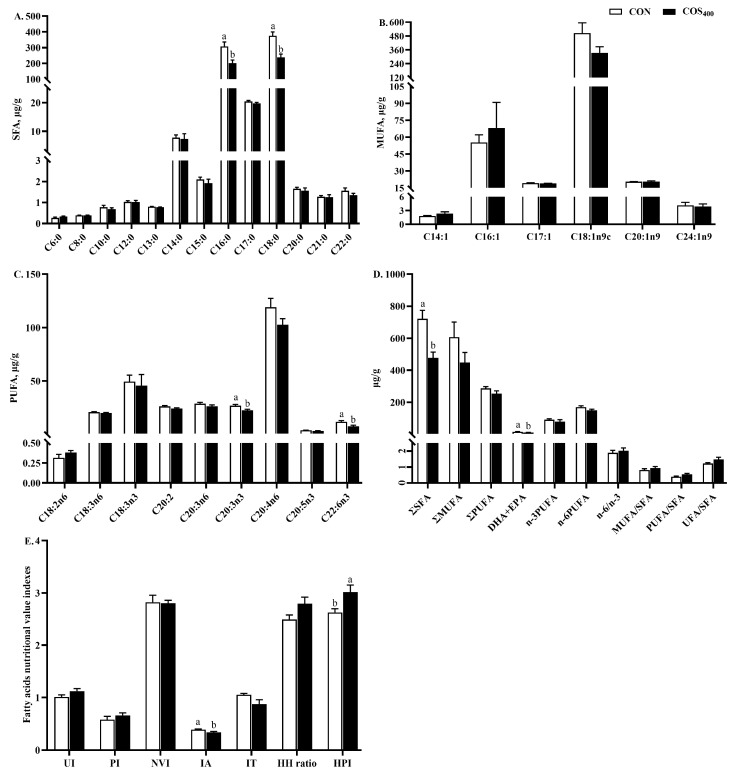
Effects of dietary chitosan oligosaccharide supplementation on fatty acid composition in the thigh muscle of broilers. Fatty acids composition (**A**–**D**) and fatty acid nutrition index (**E**). CON and COS_400_ basal diet with chitosan oligosaccharides at 0 or 400 mg/kg, respectively; the fatty acids are divided into saturated fatty acids (SFAs, including C6:0, C8:0, C10:0, C12:0, C13:0, C14:0, C15:0, C16:0, C17:0, C18:0, C19:0, C20:0, C21:0, and C22:0), monounsaturated fatty acids (MUFAs, include C14:1, C16:1, C17:1, C18:1n9c, C20:1n9, and C24:1n9), polyunsaturated fatty acids (PUFAs, including C18:2n6, C18:3n6, C18:3n3, C20:2, C20:3n6, C20:3n3, C20:4n6, C22:5n3, and C22:6n3), total n-6 PUFAs (including C18:2n6, C18:3n6, C20:3n6, and C20:4n6), and total n-3 PUFAs (including C18:3n3, C20:3n3, C22:5n3, and C22:6n3). DHA (docosahexaenoic acid) + EPA (eicosatetraenoic acid) = C22:6n3 + C20:5n3; fatty acid unsaturation index (UI) = percentage of monoenoic acid × 1 + (percentage of dienoic × 2) + (percentage of trienoic × 3) + (percentage of tetraenoic × 4) + (percentage of pentaenoic × 5) + (percentage of hexaenoic × 6); peroxidation trend index (PI) = (0.025 × percentage of monoenoic acid) + (1 × percentage of dienoyl acid) + (2 × percentage of trienoic acid) + (4 × percentage of tetraenoic acid) + (6 × percentage of pentaenoic acid) + (8 × C22:6n3, percentage of hexaenoic acid); nutrition value index (NVI) = (C18:0 + C18:1n9)/(C16:0); health-promoting index (HPI) = (ΣUFA)/(4 × C14:0 + C16:0); index of atherogenicity (IA) = (4 × C14:0 + C16:0)/(ΣMUFA + ΣPUFA); index of thrombogenicity (IT) = (C14:0 + C16:0 + C18:0)/[(0.5 × MUFA) + (0.5 × Σn-6PUFA) + (3 × Σn-3PUFA) + (Σn-3PUFA/Σn-6PUFA)]; hypocholesterolemic to hypercholesterolemic ratio (HH ratio) = (C18:1 + ΣPUFA)/(C14:0 + C16:0); and health-promoting index, (HPI) = (ΣUFA)/(4 × C14:0 + C16:0). ^a, b^ Different superscripts indicate significant difference at *p* < 0.05.

The results of lipid metabolism-related gene expression analysis of the thigh muscle showed that dietary 400 mg/kg COS resulted in better fatty acid synthesis and intramuscular preadipocyte differentiation, maturation, and proliferation. The fatty acid compositions in the CON and COS_400_ groups were measured to determine whether COS regulated the fatty acid compositions in the thigh muscle (Figure 2). Compared to the CON group, dietary 400 mg/kg COS decreased (*p* < 0.05) the content of C16:0, C18:0, C20:3n3, C20:6n3, total SFA, DHA + EPA, and IA, but increased HPI. No significant differences were observed in MUFA-related parameters.

### 3.4. Muscle Antioxidant Status

**Figure 3:**
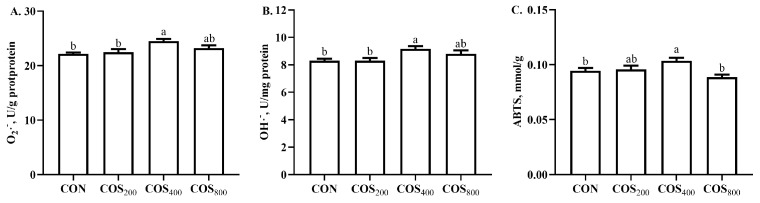
Effects of chitosan oligosaccharide supplementation on free radical scavenging activity in the thigh muscle of broilers. The free radical scavenging activity of O_2_·^−^ (**A**), OH·^−^ (**B**), and ABTS (**C**). CON, COS_200_, COS_400_, COS_800_, basal diet with chitosan oligosaccharide at 0, 200, 400, or 800 mg/kg, respectively. O_2_·^−^, oxygen radical; OH·^−^, hydroxyl radical; ABTS, 2,2′-azino-bis (3-ethylbenzothiazoline-6-sulfonate). ^a, b^ Different superscripts indicate significant difference at *p* < 0.05.

In the thigh muscle, dietary 400 mg/kg COS increased (*p* < 0.05) O_2_·^−^ and OH·^−^ scavenging activity when compared to the CON and COS_200_ groups (Figure 3), and increased (*p* < 0.05) ABTS· scavenging activity when compared to the CON and COS_800_ groups.

**Figure 4:**
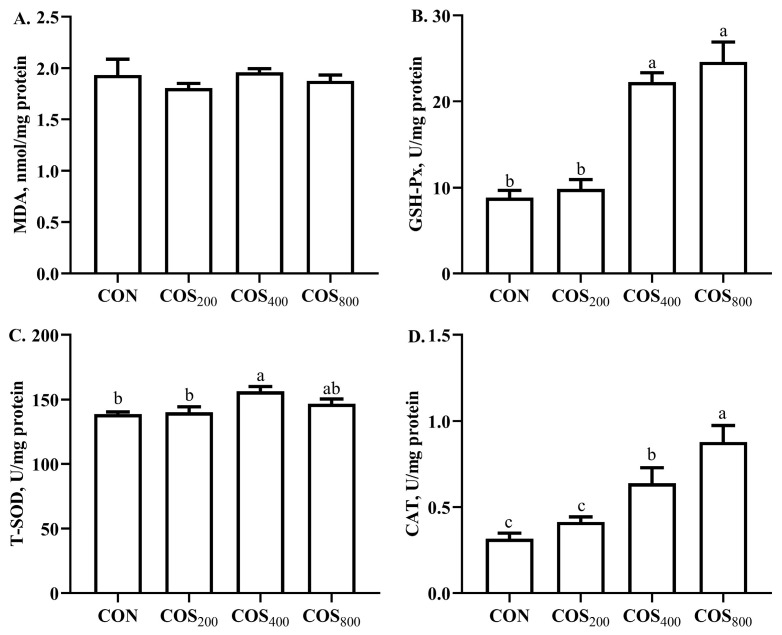
Effects of chitosan oligosaccharide supplementation on antioxidant capacity in the thigh muscle of broilers. The antioxidant capacity of MDA (**A**), GSH-Px^−^ (**B**), T-SOD (**C**), and CAT (**D**). CON, COS_200_, COS_400_, COS_800_, basal diet with chitosan oligosaccharide at 0, 200, 400, or 800 mg/kg, respectively; MDA, malondialdehyde; GSH-Px, glutathione peroxidase; T-SOD, total superoxide dismutase; CAT, catalase. ^a–c^ Different superscripts indicate significant difference at *p* < 0.05.

Dietary 400 and 800 mg/kg COS increased (*p* < 0.05) GSH-Px and CAT activity (Figure 4), and dietary 400 mg/kg COS increased (*p* < 0.05) T-SOD activity when compared to the CON and COS_200_ groups. In addition, dietary 800 mg/kg COS increased (*p* < 0.05) CAT activity when compared to the COS_400_ group.

**Figure 5:**
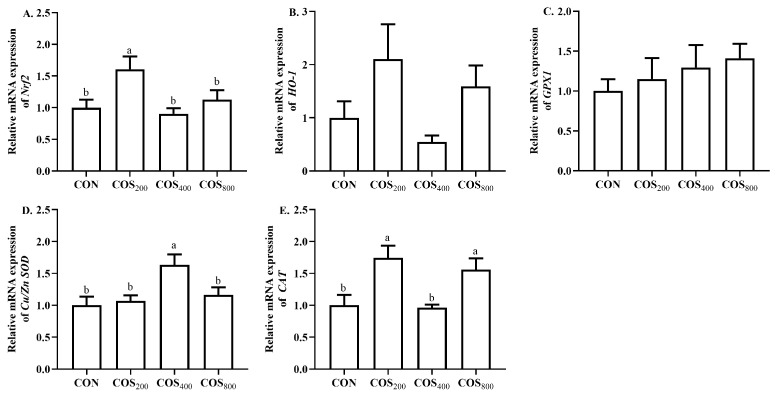
Effects of chitosan oligosaccharide supplementation on the mRNA expression of antioxidant-related gene expression in the thigh muscle of broilers. The relative mRNA expression of *Nrf2* (**A**), *HO-1* (**B**), *GPX1* (**C**), *Cu*/*Zn SOD* (**D**), and *CAT* (**E**). *Nrf2*, nuclear factor erythroid 2-related factor 2; *HO-1*, heme oxygenase 1; *GPX1*, glutathione peroxidase; *Cu*/*Zn SOD*, superoxide dismutase; *CAT*, catalase. CON, COS_200_, COS_400_, COS_800_, basal diet with chitosan oligosaccharide at 0, 200, 400, or 800 mg/kg, respectively. ^a, b^ Different superscripts indicate significant difference at *p* < 0.05.

To further investigate the effects of COS supplementation on antioxidant capacity, the mRNA expression level of antioxidant-related genes was detected (Figure 5). Dietary 200 mg/kg COS upregulated (*p* < 0.05) the mRNA expression of nuclear factor erythroid 2-related factor 2 (*Nrf2*) when compared to the other groups. Dietary 400 mg/kg COS upregulated (*p* < 0.05) the mRNA expression of superoxide dismutase (*Cu*/*Zn SOD*) when compared to the other groups. Dietary 200 and 800 mg/kg COS upregulated (*p* < 0.05) the mRNA expression of *CAT* when compared to the CON and COS_400_ groups. In addition, dietary 200 mg/kg COS upregulated (*p* < 0.05) the mRNA expression of *Nrf2* when compared to the COS_400_ and COS_800_ groups, while dietary 200 and 800 mg/kg COS upregulated (*p* < 0.05) the mRNA expression of *CAT* when compared to the COS_400_ group. There were no significant differences in the mRNA expression of heme oxygenase 1 (*HO-1*) and glutathione peroxidase (*GPX1*) among the treatments.

### 3.5. Morphological Characteristics

**Figure 6:**
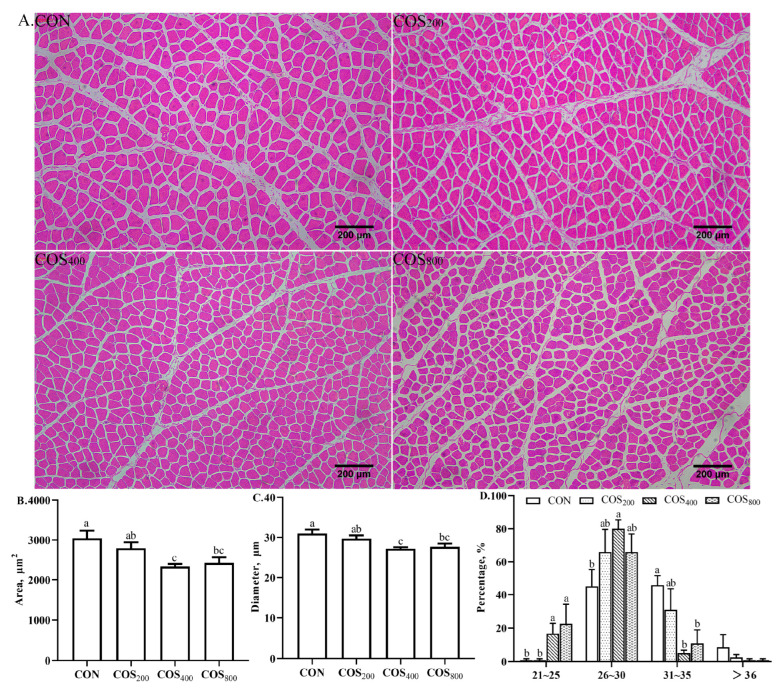
Effects of chitosan oligosaccharide supplementation on myofiber morphology characteristics in the thigh muscle of broilers. (**A**) Hematoxylin and eosin staining (10×); (**B**) myofiber cross-sectional area; (**C**) myofiber diameter; (**D**) myofiber distribution in different diameter classes (%). ^a–c^ Different superscripts indicate significant difference at *p* < 0.05.

In the thigh muscle, dietary 400 and 800 mg/kg COS decreased (*p* < 0.05) average myofiber CSA and diameter when compared to the CON group (Figure 6). Meanwhile, the COS_400_ and COS_800_ groups exhibited more myofibers ranging from 21 to 25 μm in diameter than the CON and COS_200_ groups. The COS_400_ group exhibited more myofibers ranging from 26 to 30 μm in diameter than the CON group. The COS_400_ and COS_800_ groups exhibited fewer myofibers ranging from 31 to 35 μm in diameter than the CON group. The abundance of large diameter myofibers (>36 μm) was consistent across all groups.

### 3.6. Myogenic Regulatory Factors, Myofiber Transformation-Related Gene Expression

**Figure 7:**
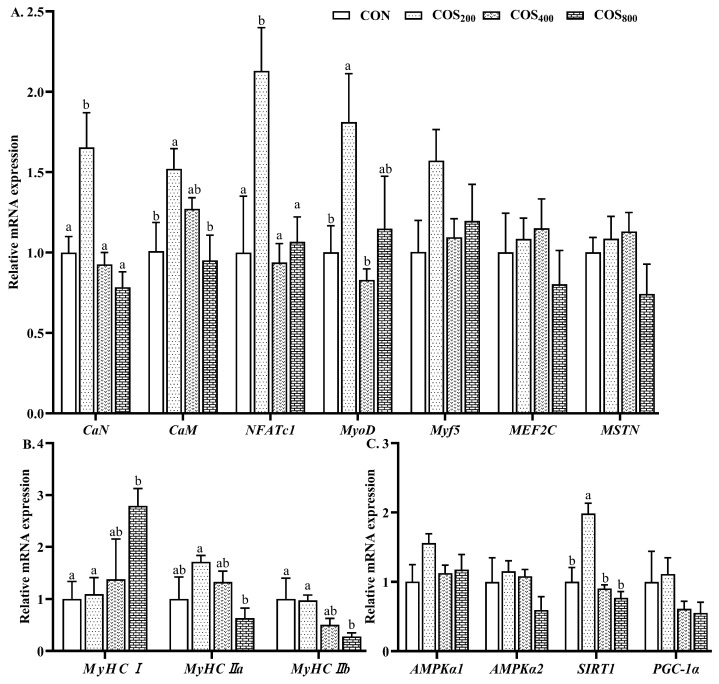
Effects of chitosan oligosaccharide supplementation on myogenic regulatory factors, muscle fibers, and fiber transformation-related gene expression in the thigh muscle of broilers. Relative mRNA expression of myogenic regulatory factors related genes (**A**), different muscle fibers related genes (**B**), and muscle fiber transformation related genes (**C**). CON, COS_200_, COS_400_, COS_800_, basal diet with chitosan oligosaccharide at 0, 200, 400, or 800 mg/kg, respectively. *CaN*, calcineurin; *CaM*, calmodulin; *NFATc1*, nuclear factor of activated T cells c1; *MyoD*, myogenic differentiation antigen; *Myf5*, myogenic regulatory factor 5; *MEF2C*, myocyte enhancer factor 2C; MSTN, myostatin; *MyHC*, myosin heavy chain; *AMPK*, adenosine 5′-monophosphate-activated protein kinase; *SIRT1*, sirtuin 1; *PGC-1α*, peroxisome proliferator-activated receptor-γ coactivator-1α. ^a, b^ Different superscripts indicate significant difference at *p* < 0.05.

In the thigh muscle, dietary 200 mg/kg COS upregulated (*p* < 0.05) the mRNA expression of calcineurin (*CaN*), nuclear factor of activated T cells c1 (*NFATc1*), and sirtuin 1 (*SIRT1*) when compared to the other groups (Figure 7). Dietary 200 mg/kg COS upregulated (*p* < 0.05) the mRNA expression of calmodulin (*CaM*) when compared to the CON and COS_800_ groups. Dietary 200 mg/kg COS upregulated (*p* < 0.05) the mRNA expression of myogenic differentiation antigen (*MyoD*) when compared to the CON and COS_400_ groups. Dietary 800 mg/kg COS upregulated (*p* < 0.05) the mRNA expression of *MyHCI*, while downregulating (*p* < 0.05) the mRNA expression of *MyHCIIb* when compared to the CON and COS_200_ groups. In addition, dietary 800 mg/kg COS downregulated (*p* < 0.05) the mRNA expression of *MyHCIIa* when compared to the COS_200_ group. There were no significant differences in the mRNA expression of myogenic regulatory factor 5 (*Myf5*), myocyte enhancer factor 2C (*MEF2C*), myostatin (*MSTN*), or peroxisome proliferator-activated receptor-γ coactivator-1α (*PGC-1α*) among the treatments.

## 4. Discussion

As a nutritious protein source, the demand for high-quality chicken meat products is increasing with the desire for health and a higher quality of life. Meat quality is primarily evaluated by various factors, such as pH value, meat color, water holding capacity, tenderness, intramuscular fat (IMF) content, chemical composition, and nutritional value [26]. In this study, dietary 200 and 400 mg/kg COS decreased shear forces. A study in Huoyan geese also indicated that dietary 400 mg/kg COS decreased shear forces [13]. Shear force is a meat trait associated with consumer satisfaction with eating quality [27]. The decreased shear force suggested a decrease in firmness and improvement in tenderness and meat quality in the thigh muscle of broilers. In general, IMF and crude protein contents are associated with meat flavor and nutritional value [28]. In this study, dietary 400 mg/kg COS increased crude protein content, suggesting that COS supplementation improved nutritional value in the thigh muscle. IMF content and fatty acid composition are regarded as the key indicators of meat quality, especially eating quality [13,29]. IMF content consists of an increase in adipocyte number (hyperplasia) and volume (hypertrophy). Previous studies indicated that adipocyte differentiation and proliferation and lipid deposition are controlled by *PPARγ*, *C/EBPα*, and *C/EBPβ* [30]. In this study, dietary 400 and 800 mg/kg COS upregulated the expression of *PPARγ*, and dietary 200 mg/kg COS upregulated the expression of *C/EBPα*, which suggested enhancing adipocyte differentiation and maturation. The increased IMF content is associated with tenderness, and the changes in expression of lipid metabolism-related genes contribute differently to meat quality [31]. Therefore, the upregulated expression of *PPARγ* and *C/EBPα* may partly explain the decreasing shear force. For chicken meat, the fatty acid composition and proportion are highly associated with its taste and nutritional value, and probably influence further storage and processing [23]. Excessive SFA consumption results in higher cholesterol levels, subsequently increasing the incidence of cardiovascular disease [32]. Excessive n-6 PUFA consumption increases in the incidence of obesity, inflammation, and cancer [33]. An n-6:n-3 PUFA ratio ranging from 1.1 to 2.1 is associated with health benefits [34]. It is believed that higher n-3 PUFA and lower SFA contents will enhance chicken meat nutritional value and health benefits. In this study, the predominant SFAs detected in the thigh muscle were C16:0 and C18:0, which together accounted for over 90% of the SFAs. The predominant MUFA detected in the thigh muscle was C18:1cis9, which accounted for over 80% of the MUFAs. The predominant PUFA detected in the thigh muscle was C20:4n6, which accounted for over 40% of the PUFAs. Dietary 400 mg/kg COS decreased C16:0, C18:0, and total SFA contents when compared to the CON group, consistent with the mRNA expression of lipid metabolism-related genes. The decreased C16:0, C18:0, and total SFA contents in the COS_400_ group can be partially explained by the upregulated expression of *PPARα*, which is involved in fatty acid β-oxidation. Additionally, dietary 400 mg/kg COS decreased IA but increased HPI. The IA reflects the atherogenic and thrombogenic potential of fatty acids [35]. However, the HPI is opposite to IA; the higher the HPI value, the better the nutritional value of chicken meat [23]. These results suggest that dietary 400 mg/kg COS improved nutritional value by decreasing C16:0, C18:0, and total SFA contents, subsequently decreasing IA and increasing HPI. Similar results have also been reported by Miao et al. [13], who reported that dietary 200 mg/kg COS decreased C16:0, C18:0, and total SFA contents in the breast muscle of Huoyan geese. Zhou et al. [36] also reported that dietary 200 and 400 mg/kg COS decreased C14:0, C16:0, C18:0, and total SFA contents in the breast muscle of broilers. On the other hand, the results indicated that dietary 400 mg/kg COS decreased the C20:3n3 and C22:6n3 contents in the thigh muscle when compared to the CON group. Both C20:3n3 and C22:6n3 are essential fatty acids which can only be obtained from the daily diet. It is unclear how COS supplementation decreased C20:3n3 and C22:6n3 contents in the thigh muscle, and the reasons needs to be investigated further.

Chicken meat is susceptible to oxidation due to its high PUFA content; therefore, the meat quality is highly associated with antioxidant capacity [37]. The existing literature indicates that COS has excellent antioxidant and free radical scavenging capacity [6]. In this study, dietary 400 mg/kg COS improved the free radical scavenging capacity of O_2_·^−^, OH·^−^, and ABTS· in the thigh muscle. In consistent with our results, the existing literature reported that dietary 300, 600, and 900 mg/kg COS enhanced the O_2_·^−^, OH·^−^, and ABTS· scavenging capacity in the breast and thigh muscle of yellow-feathered broilers [12]. COS possesses functional amino groups, a carboxyl group, and primary and secondary hydroxyl groups at the C-2, C-3, and C-6 positions; these functional groups may explain the beneficial effects on free radical scavenging [38]. Antioxidant enzyme activity directly reflects the muscle antioxidant capacity. In this study, dietary 400 and 800 mg/kg COS increased GSH-Px and CAT activity, and dietary 400 mg/kg COS increased T-SOD activity in the thigh muscle. Consistent with our results, Lan et al. [12] reported that dietary 300, 600, and 900 mg/kg COS increased the GSH-Px, SOD, and CAT activity in the thigh muscle of yellow-feathered broilers. The antioxidant enzyme activity is highly associated with the antioxidant-related gene expression. The existing literature indicated that COS could activate the erythroid 2-related factor 2–antioxidant responsive element (Nrf2-ARE) signaling pathway to upregulate the expression of antioxidant-related genes [8]. In this study, dietary 200 mg/kg COS upregulated the gene expression of *Nrf2*, dietary 400 mg/kg COS upregulated the gene expression of *Cu*/*Zn SOD*, and dietary 200 and 800 mg/kg COS upregulated the gene expression of *CAT* in the thigh muscle. Similar to our results, Lan et al. [12] reported that dietary 600 mg/kg COS upregulated the expression of *Nrf2* and *HO-1*, dietary 900 mg/kg COS upregulated the expression of *SOD*, and dietary 300 and 600 mg/kg COS upregulated the expression of *GPX1* in thigh muscle. Chang et al. [8] also reported that dietary 400 mg/kg COS upregulated the gene expression of *HO-1* and *GPX1* in the breast muscle of heat-stressed yellow-feathered broilers. Obviously, COS enhanced thigh muscle antioxidant capacity by enhancing free radical scavenge ability, stimulating antioxidant enzyme activity, and upregulating antioxidant-related gene expression. These results suggest that the best beneficial effects of COS on antioxidant capacity of thigh muscle appear when its supplemental level is 400 mg/kg.

Myofibers account for 70–90% of the volume of skeletal muscle. The composition and proportion of myofiber types directly affect muscle biochemical, physiological, structural, and morphological characteristics, and directly determine muscle volume and meat quality [39,40]. The shear force of thigh muscle is negatively correlated with myofiber diameter, and smaller myofiber CSA and diameter are highly correlated with lower shear force and better tenderness [41]. In this study, dietary 400 mg/kg COS decreased the myofiber average diameter and CSA, and dietary 400 and 800 mg/kg COS increased the relative abundance of myofibers with a diameter ranging from 21 to 25 μm and 26 to 30 μm but decreased the relative abundance of myofibers with a diameter ranging from 31 to 35 μm when compared to the CON group. Skeletal muscle contains three main fiber types; type I fibers (*MyHCI*) contain more mitochondria and aerobic metabolic enzymes, type IIa fibers (*MyHCIIa*) contain aerobic metabolic enzymes and glycolytic enzymes, and type IIb fibers (*MyHCIIb*) contain fewer mitochondria and more glycogen and glycolytic enzymes [19]. The composition and proportion of these myofiber types directly influences meat quality, including water holding capacity, meat color, pH, tenderness, juiciness, and flavor [9,42]. Skeletal muscle rich in the *MyHCI* type has better aerobic metabolism, as well as better appearance, eating quality, and flavor [43]. However, skeletal muscle rich in the *MyHCIIb* type can accelerate postmortem muscle glycolysis, and subsequently decrease pH, water holding capacity, and flavor [44]. In this study, dietary 800 mg/kg COS upregulated the mRNA expression of *MyHCI* while downregulating the mRNA expression of *MyHCIIb*. These results suggest that dietary 800 mg/kg COS promotes myofiber type transformation from *MyHCIIb* to *MyHCI*.

The number of muscle fibers formed before birth and myofiber development are mainly due to myofiber hypertrophy and type transformation [9,43]. The existing literature shows that myogenic regulatory factors, including *MyoD*, *Myf5*, and *MEF2C*, play vital roles in myogenesis and myogenic differentiation [9]. In this study, dietary 200 mg/kg COS upregulated the expression of *MyoD*, suggested that dietary 200 mg/kg COS promoted myogenesis in the thigh muscle, and subsequently affected myofiber characteristics [45]. At birth, muscle is composed of type I and IIa myofibers, while the proportion of type IIb myofibers increases during growth [43]. The increased proportion of type IIb myofibers accompanies a decreasing proportion of type I and IIa myofibers. Myofiber types are dynamic structures and exhibit high plasticity capacity, undergoing type shifts following an obligatory pathway I ↔ IIa ↔ IIb [46]. The existing literature indicates that *CaN* exhibits an important role in myofiber transformation [47]. *CaN* can selectively enhance the promoter activity of *MyHCI* [48]. *NFATc1* is the main substrate of *CaN*, and the activated *NFATc1* regulates type I fiber-related gene transcription. Meanwhile, *MEF2C* is another substrate of *CaN*, and the activated *NFATc1* and *MEF2C* regulate the expression of *Myf5* [49]. In this study, dietary 200 mg/kg COS upregulated the expression of *CaN*, *CaM*, *NFATc1*, and *MyoD*. These results suggest that the myofiber transformation was associated with the upregulation of *CaN*, *CaM*, *NFATc1*, and *MyoD*. Furthermore, *AMPK* also plays a critical role in myofiber transformation. *SIRT1* and *PGC-1α* are the substrates of *AMPK*. When activated by *AMPK*, *SIRT1* and *PGC-1α* promote myofiber transformation from type IIb to type I fibers [18]. In this study, dietary 200 mg/kg COS upregulated the expression of *SIRT1*, suggesting that the *AMPK–SIRT1* signaling pathway is another way to regulate myofiber transformation. Obviously, these results are not consistent; dietary 200 mg/kg upregulated the expression of *CaN*, *CaM*, *NFATc1*, *MyoD*, and *SIRT1*, but dietary 800 mg/kg upregulated the expression of *MyHCI* while downregulating the expression of *MyHCIIb*. The reasons were unclear and need to be investigated further.

## 5. Conclusions

Dietary COS supplementation has beneficial effects on meat quality, nutritional value, antioxidant capacity, and myofiber characteristics in the thigh muscle of broilers. These conclusions are supported by the findings that dietary COS supplementation decreases shear force and enhances nutritional value through increased crude protein content, a desirable fatty acid composition, lower IA, and higher HPI. Dietary COS supplementation regulates fatty acid composition by regulating the expression of *PPARα*, *PPARγ*, and *C*/*EBPα*. Furthermore, dietary COS supplementation-related improvements in meat quality may be associated with the improvement of antioxidant capacity and myofiber transformation to more oxidative muscle fibers in the thigh muscle. The myofiber transformation is associated with the upregulated gene expression of *CaN*, *NFATc1*, *MyoD*, and *SIRT1.* Combining all the parameters we investigated, the beneficial effects on meat quality were more apparent when the COS supplemental level was 400 mg/kg.

## Figures and Tables

**Table 1:** Effects of dietary chitosan oligosaccharide supplementation on meat quality and nutrient composition in the thigh muscle of broilers.

Items ^1^	CON	COS_200_	COS_400_	COS_800_	SEM ^2^	*p*-Value
pH_45min_	6.46 ± 0.05	6.48 ± 0.06	6.45 ± 0.06	6.45 ± 0.08	0.06	0.9880
pH_24h_	6.24 ± 0.04	6.29 ± 0.06	6.17 ± 0.03	6.14 ± 0.23	0.12	0.8075
Lightness, L*	53.63 ± 1.54	54.05 ± 0.98	54.35 ± 0.91	54.58 ± 1.60	1.30	0.9595
Redness, a*	4.49 ± 0.11	4.56 ± 0.13	4.86 ± 0.11	4.85 ± 0.20	0.14	0.1692
Yellowness, b*	6.88 ± 0.08	6.76 ± 0.15	6.81 ± 0.20	6.75 ± 0.18	0.16	0.9382
Drip loss, %	6.60 ± 0.44 ^ab^	6.24 ± 0.25 ^b^	6.16 ± 0.24 ^b^	7.42 ± 0.41 ^a^	0.35	0.0472
Cooking loss, %	25.23 ± 1.76	23.74 ± 1.46	25.52 ± 2.16	27.19 ± 1.90	1.847	0.6277
Shear force, N	25.20 ± 1.43 ^a^	19.35 ± 0.79 ^bc^	16.83 ± 1.19 ^c^	22.03 ± 0.98 ^ab^	1.12	0.0003
Dry matter, %	80.90 ± 0.13 ^a^	79.84 ± 0.09 ^b^	80.72 ± 0.12 ^a^	79.80 ± 0.06 ^b^	0.10	<0.0001
Crude protein, %	23.91 ± 0.59 ^b^	21.81 ± 1.37 ^b^	28.64 ± 2.23 ^a^	21.63 ± 1.21 ^b^	1.47	0.0102
Crude lipid, %	2.17 ± 0.15	2.18 ± 0.17	2.13 ± 0.09	2.31 ± 0.09	0.12	0.7508

^1^ CON, COS_200_, COS_400_, COS_800_, basal diet with chitosan oligosaccharide at 0, 200, 400, or 800 mg/kg, respectively. ^2^ SEM, standard error of mean. ^a–c^ Different superscripts indicate significant difference at *p* < 0.05.

## Data Availability

The data presented in this study are available on request from the corresponding author.

## Supplementary Materials

The following supporting information can be downloaded at: https://www.mdpi.com/article/10.3390/antiox13030366/s1, Table S1: Ingredient composition and nutrient content of diets; Table S2: The commercial kit information; Table S3: The primer sequences.
