# Supplementary material for: Dietary Chitosan Oligosaccharide Supplementation Improves Meat Quality by Improving Antioxidant Capacity and Fiber Characteristics in the Thigh Muscle of Broilers
Source: Antioxidants (Basel). 2024 Mar 18;13(3):366. doi: 10.3390/antiox13030366 (PMC10967738; doi:10.3390/antiox13030366)
Supplement: Supplementary file 1 [file antioxidants-13-00366-s001.zip › antioxidants-2915612-supplementary.pdf]

antioxidants

MDPI

Table S1. Ingredient composition and nutrient content of diets

Item	Day 1 to 22	Day 22 to 42
Ingredients, %		
Corn	54.57	62.44
Soybean meal, 48% crude protein	29.95	25.58
Corn gluten meal, 60% crude protein	5.90	3.30
Soybean oil	5.50	4.89
Tricalcium phosphate	2.46	2.29
Limestone	0.89	0.75
Salt	0.20	0.20
DL-Met, 88%	0.07	0.07
L-Lys·HCl (78.4%)	0.06	0.08
Vitamin premix2	0.20	0.20
Mineral premix3	0.20	0.20
Calculated composition		
Metabolic energy, MJ/kg	12.95	12.74
Crude protein, %	21.89	18.90
Calcium, %	1.05	0.96
Lys, %	1.12	1.01
Met + Lys, %	0.90	0.86
Available phosphorus, %	0.81	0.73
Analyzed composition, %		
Crude protein	21.12	20.02
Calcium	1.03	0.95
Met + Lys	0.89	0.87
Available phosphorus	0.44	0.42

[1] Provided per kilogram of complete diet: 12,8000 IU vitamin A, 1,600 IU vitamin D_3, 60 IU vitamin E, 1.6mg vitamin K_3, 0.12mg biotin, 50mg choline, 1.2mg folic acid, 32mg Nicotinic acid, 16mg pantothenic acid, 4.8mg riboflavin, 2.4mg thiamine (B_1), 3.2mg vitamin B_6, and 0.03mg vitamin B_{12}.

[2] Provided per kilogram of diet: Mg, 79mg as manganese oxide; Zn, 60mg as zinc oxide; Cu,100mg as copper sulfate; Fe, 120mg as iron sulfate; I, 0.96mg as potassium iodine; Co, 0.16mg as cobalt sulfate and Se, 0.24mg as sodium selenite.

Table S2. The commercial kits information

Index[1]	Name of kits	Cat. No.
MDA	Malondialdehyde (MDA) assay kit (TBA method)	A003-1-2
CAT	Catalase (CAT) assay kit (Visible light)	A007-1-1
GSH-Px	Glutathione peroxidase (GSH-PX) assay kit (colorimetry)	A005-1-2
T-SOD	Total Superoxide Dismutase (T-SOD) assay kit (Hydroxylamine method)	A001-1-2
$O_2{\cdot}^-$	Inhibition and produce superoxide anion assay kit	A082-3-1
$OH{\cdot}^-$	Hydroxyl Free Radical assay kit	A018-1-1
ABTS·	ABTS free radical scavenging capacity assay kit	A015-2-1

[1] MDA, malondialdehyde; GSH-Px, glutathione peroxidase; T-SOD, total superoxide dismutase; CAT, catalase; $O_2{\cdot}^-$, oxygen radical; $OH{\cdot}^-$, hydroxyl radical; ABTS·, 2, 2′-azino-bis (3-ethylbenzothiazoline-6-sulfonate).

Table S3. The primer sequences

Gene[1]	Accession NO.	Primer sequence (5′ to 3′)
β-actin	NM_205518.1	F: ATCCGGACCCTCCATTGTC<br>R: AGCCATGCCAATCTCGTCTT
SREBP-1c	XM_046927256.1	F: CATCCATCAACGACAAGATCGT<br>R: CTCAGGATCGCCGACTTGTT
ACC	XM_046929960.1	F: GCTTCCCATTTGCCGTCCTA<br>R: GCCATTCTCACCACCTGATTACTG
FAS	NM_205155.4	F: TTTGGTGGTTCGAGGTGGTA<br>R: CAAAGGTTGTATTTCGGGAGC
PPARα	XM_046906400.1	F: TTTAACGGAGTTCCAATGGC<br>R: AACCCTTACAACCTTCACAAGC
CPT-1	XM_046918285.1	F: TAGAGGGCGTGGACCAATAA<br>R: TGGGATGCGGGAGGTATT
PPARγ	NM_001001460	F: CAGTGGATCTGTCTGCGATG<br>R: CTTTGGCAATCCTGGAGCTTG
C/EBPα	NM_001031459	F: GACATCTGCGAGAACGAGCA<br>R: GCATGCCGTGGAAATCGAAA
C/EBPβ	NM_205253.2	F: GCCGCCCGCCTTTAAA<br>R: CCAAACAGTCCGCCTCGTAA
Nrf2	NM_205117.1	F: GAGAGCGGCAGCAAGATGACAG<br>R: CAGCCAGGTGTGGTTGTAGAAGTC
HO-1	XM_205344.1	F: GATGTGCGGATACCTGAAGC<br>R: AGGGATGCCAACATGACTGA
GPX1	NM_000581.4	F: GCATTGTTTCTAGCTTGCGGT<br>R: TCCTCCTGAGAACGGACTGT
Cu/Zn SOD	NM_205064.1	F: ACCCTTTTGCCTTGGAAACT<br>R: TTGAGATGTTTGCGTGAAG
CAT	NM_001031215.2	F: GGGGAAGAGCGCGAGACGGA<br>R: CGTCATTGTATGGGTGATGG
CaN	XM_025149952.1	F: TTGTCTGATGGAGATCATGGCTTC<br>R: TGCTTGCCTTCAGGATTAAAGTGAG
CaM	NM_205005.2	F: GATGAGATCCTGAGAGTGGTGGAC<br>R: TCATCAGGTAAGGTGGCACAA
NFATc1	XM_025147636.1	F: GGTCTTCCGAGTTCACATCC<br>R: TCTCCACCAGAGGCAGTTCT
MyoD	NM_204214.2	F: AACCTGAGTGACAGTGGAGC<br>R: TCTTGGAGCTTGGCTGAACG
Myf5	NM_001030363.1	F: GCAGCCACTATGAGGGAGAG<br>R: GATGTACCTGATGGCGTTCC
MEF2C	XM_025144794.1	F: CCATCAGCCATCTCAACAAC<br>R: CAGCCAGTCACAGAACCAAG
MSTN	NM_001001461.2	F: GCCTGGAACAAGCACCTAAC<br>R: GCTACTGTCGTCCCTCTGGA
MyHC I	XM_046930560.1	F: ATCACGAGCCCTGAAACCAA<br>R: GGCTGCAAAATGCTGGAAAA
MyHC IIa	NM_204228.4	F: ACTTCTATGGCAGCAACT<br>R: AATAGCGGGTGTAGGC
MyHC IIb	NM_001013396.2	F: TACGGTTCTCCACTGTTGCTG<br>R: TGGATGAAGGATGGAAACAAC
AMPKα1	NM_001039603.2	F: ATCTGTCTCGCCCTCATCCT

		R: CCACTTCGCTCTTCTTACACCTT
AMPKα2	NM_001039605.2	F: CCAGCGAGTTCTACCTAGCCT
		R: TGCCTTGGGACTGTCTGCA
SIRT1	XM_046920057.1	F: CACGCCTTGCTGTAGACTTCC
		R: ATGAACTTGTGGCAGAGAGATGG
PGC-1α	XM_015285697.2	F: CGTGGAGCAATAAAGCGAAG
		R: TCTGAGGAGGGTCATCGTTC

[1] *SREBP-1c*, sterol regulatory element-binding protein-1c; *ACC*, acetyl-CoA carboxylase; *FAS*, fatty acid synthase; *PPAR*, peroxisome proliferator-activated receptor; *CPT-1*, carnitine palmitoyl transferase 1; *C/EBP*, CCAAT/enhancer binding protein; *Nrf2*, nuclear factor erythroid 2-related factor 2; *HO-1*, neme oxygenase 1; *GPX1*, glutathione peroxidase; *Cu/Zn SOD*, superoxide dismutase; *CAT*, catalase; *CaN*, calcineurin; *CaM*, calmodulin; *NFATc1*, nuclear factor of activated T cells c1; *MyoD*, myogenic differentiation antigen; *Myf5*, myogenic regulatory factors 5; *MEF2C*, myocyte enhancer factor 2C; MSTN, myostatin; *MyHC*, myosin heavy chain; *AMPK*, adenosine 5′-monophosphate-activated protein kinase; *SIRT1*, sirtuin 1; *PGC-1α*, peroxisome proliferator-activated receptor-γ coactivator-1α.
